# First Symptoms of Primary Progressive Aphasia and Alzheimer's Disease in Brazilian Individuals

**DOI:** 10.3389/fneur.2021.628406

**Published:** 2021-06-11

**Authors:** Talita Gallas dos Reis, Thais Helena Machado, Paulo Caramelli, Francisco Scornavacca, Liana Lisboa Fernandez, Bárbara Costa Beber

**Affiliations:** ^1^Departamento de Fonoaudiologia, Universidade Federal das Ciências da Saúde de Porto Alegre, Porto Alegre, Brazil; ^2^Departamento de transtornos cognitivos e demências, Universidade Federal de Minas Gerais, Belo Horizonte, Brazil; ^3^Departamento de Clínica Médica—Neurologia, Universidade Federal de Minas Gerais, Belo Horizonte, Brazil; ^4^Departamento de Pediatria, Universidade Federal das Ciências da Saúde de Porto Alegre, Porto Alegre, Brazil; ^5^Departamento de Ciências Básicas da Saúde, Universidade Federal das Ciências da Saúde de Porto Alegre, Porto Alegre, Brazil

**Keywords:** aphasia, primary progressive aphasia, Alzheimer's disease, differential diagnosis, signs and symptoms, language

## Abstract

**Background:** Primary Progressive Aphasia (PPA) is characterized by progressive language impairment due to focal degeneration of brain areas related to linguistic processing. The detection and differential diagnosis of PPA can be difficult with clinical features that may overlap with features of other neurological conditions, such as Alzheimer's disease (AD). The scientific production on PPA in Latin American patients is still scarce. This study investigated the first symptoms in a Brazilian sample of patients with PPA in comparison with AD patients.

**Method:** We compared the first symptoms reported by caregivers of people with PPA (*n* = 20; semantic variant *n* = 8, non-fluent variant *n* = 7, logopenic variant *n* = 3, and unclassified cases *n* = 2) and AD (*n* = 16). Data were collected through the application of a structured questionnaire that was presented in an interview format to the caregiver who knew the patient best.

**Results:** Anomia, paraphasias and motor speech difficulties were the first symptoms capable of differentiating patients with PPA from those with AD, while memory was exclusive of AD. Among the PPA variants, anomia was the initial symptom associated with the semantic variant, while motor speech difficulties were associated with the non-fluent variant. The results are discussed considering the unique cultural and sociodemographic characteristics of this studied population.

**Conclusion:** This study demonstrated that some of the initial symptoms of PPA patients may be unique to clinical variants of PPA and of AD, and their investigation may be useful for the early and differential diagnosis of this population.

## Introduction

Primary progressive aphasia (PPA) is a neurological syndrome characterized by a progressive and prominent language impairment. It occurs due to neurodegenerative processes in the frontotemporal regions, predominantly in the left hemisphere (1–3). Language impairment should appear relatively isolated, without equivalent changes in other cognitive domains, in addition to the indication of a neurodegenerative condition, in order to confirm a diagnosis of PPA (1, 4, 5). Aphasia should be the most prominent deficit during the early stages of the disease (1). For this reason, the first symptoms must be investigated and described in order to differentiate PPA from other neurological disorders that have a different symptomatic picture in the early stages of the disease, and to perform the differential diagnosis of PPA variants.

There are three variants of PPA, which have specific characterization and diagnostic criteria (1, 3, 6, 7). Semantic variant (svPPA): characterized by fluent spontaneous speech, but with recurrent episodes of anomia and difficulty in understanding isolated words. Subjects may have verbal and semantic paraphasias, generalizations, omissions, in addition to reading and writing difficulties. The clinical condition is due to the involvement of the anterior temporal areas, which may occur in both cerebral hemispheres. Non-fluent/agrammatic variant (nfvPPA): mainly characterized by non-fluent oral expression, and may include apraxia of speech and/or agramatism, with the production of simple and short sentences, slowed speech, errors in articulatory movements, changes in prosody and substitutions of speech sounds. These symptoms result from the involvement of fronto-insular areas of the left hemisphere. Logopenic variant (lvPPA): characterized by difficulty in repeating sentences and finding words at the time of oral communication, including phonological errors in speech. The symptoms in this variant are due to a neurodegeneration at the left temporoparietal junction.

In turn, Alzheimer's disease (AD) is a neurodegenerative disease often diagnosed based on clinical symptoms, which gradually worsens cognitive and behavioral domains, such as learning and memory (8). The main clinical criteria for the diagnosis of dementia due to AD include cognitive and/or behavioral changes that impact the functioning of daily activities and represent a decline from previous levels of functioning (9). The deficits should occur at least in two domains, such as impaired ability to remember new information, impaired reasoning or changes in personality and behavior (9). Common symptoms of AD include impaired ability to acquire or recall new information; impaired judgment and handling of complex activities; involvement of visuospatial skills; involvement of language domains; behavioral changes, such as apathy, hyperactivity (agitation and irritability), psychosis (delusions and hallucinations), and affective symptoms (depression and anxiety) (8, 9).

The characterization of PPA variants can easily be confused with the findings of other neurological disorders, especially with AD. Many cases of PPA are believed to be underdiagnosed, while others still remain without a closed diagnosis or with a long delay to completion (10). Studies have reported that the lvPPA may appear as an initial symptom of AD in atypical cases, being recognized as one of the non-amnestic variants of AD (6). The same occurs with semantic and non-fluent variants, that are mistakenly diagnosed as the behavioral variant of frontotemporal dementia (FTD) (11) without an adequate and accurate characterization. These diagnostic mistakes can be explained both by the common symptomatic characteristics, but also by the similar neuropathological findings of both syndromes.

Studies report that FTD in general (including PPA) in low- and middle-income countries, such as Brazil, have a late diagnosis when compared to AD (12). The delay in receiving the correct diagnosis may be related to the patients' delay in seeking medical care, the delay in the Brazilian public health system in offering care, or even to the difficulties in reaching the correct diagnosis. There is evidence that these patients suffer from diagnostic errors due to the clinicians' difficulty in differentiating the types of dementia during initial manifestations (12). One previous study conducted in Brazil already reported the need for a careful investigation of the first symptoms (12). An in-depth investigation of the initial symptoms is believed to be even more important when patients come to the referral centers at later stages of the disease.

Given these diagnostic difficulties, studies that seek differential diagnoses and characterizations of PPA compared to other disorders, such as AD, are helpful for the accuracy of diagnosis and the best clinical management of these individuals. An important alternative would be the neuropsychological and the speech/language assessments to define the appropriate classification of PPA subtypes, in order to differentiate it from other neurological disorders (11, 13, 14).

However, it is also essential to perform a clinical examination and a comprehensive anamnesis in order to investigate the occurrence of the first symptoms presented by the patient. In addition, language plays a central role in the management of PPA and, therefore, studies that investigate how the syndrome manifests itself in speakers of different languages are of great relevance. Given this context, this study aims to describe the early symptoms of patients with the three variants of PPA, compared with patients with AD, in a sample of Brazilian Portuguese speakers.

## Materials and Methods

### Participants

This is a quantitative, descriptive and cross-sectional study.

The study included a convenience sample of patients with an established diagnosis of PPA (1) or AD (15), according to current diagnostic criteria, who consented to participate in the study by signing the Informed Consent Form (ICF) by the guardian, and who had a family member or caregiver who was familiarized enough with the patient to answer the study questionnaire. The study excluded subjects who did not agree to participate and those who did not have a family member or close caregiver to answer the questionnaire.

All participants were diagnosed by a neurologist who considered information from an interview with patient and caregiver; physical examination; neuropsychological and speech and language assessment (this only for patients in suspicion of PPA); blood tests; and neuroimaging tests (magnetic resonance imaging—MRI). For some patients, cerebrospinal fluid examination with dosage of biomarkers of AD and functional neuroimaging tests (FDG-PET or SPECT) were also performed. Our sample consisted of patients from both the public and private health systems. The Brazilian public health system does not cover the costs of the cerebrospinal fluid examination AD biomarkers or functional neuroimaging, so such tests were only performed by patients who could afford to pay for these tests privately or had health insurance to cover their costs. A few patients from the public universities had results of AD biomarkers in the cerebrospinal fluid as part of other research protocols.

The study included patients from the Neurology Outpatient Clinic of the Irmandade Santa Casa de Misericórdia de Porto Alegre (ISCMPA), and from the Behavioral and Cognitive Neurology Outpatient Clinic of the Hospital das Clínicas of the Universidade Federal de Minas Gerais (HC-UFMG), in Belo Horizonte.

This study was conducted in line with local ethical standards and was approved by the Research Ethics Committee of the ISCMPA (under no. 3,117,790), and also by the Research Ethics Committee of the HC-UFMG ethics committee (under no. 2,018,855).

### Procedures

Data were collected through a structured questionnaire (Appendix 1) prepared by the researchers, which was conducted as an interview with the caregiver who had the best knowledge about the patient. The questionnaire was applied in person, or by a previously scheduled telephone call. Participants were informed about the study procedures, then read and signed the informed consent form, and finally answered the interview. When made by telephone call, the ICF was read and agreed to via an online document.

The questionnaire consisted of closed and open questions about personal and sociodemographic information (of patient and caregiver), clinical data, and the description of the first symptoms of the disease presented by the patient. The first symptoms were collected first through an open question, where the respondent had to explain with his/her words how the disease started. The next question was a closed question with a list of symptoms that was read to the respondent and he/she had to confirm if those symptoms occurred at the very beginning of the disease or not. We decided to present this list of symptoms to complement the description made by the respondent in the open question, in case he/she has forgotten any important symptom. This list of symptoms was created by the researchers in order to present the most important symptoms for the detection of PPAs and AD, in a brief way and using a plane language for the participants. The answers in the open question were transcribed and grouped into categories decided upon by consensus by the researchers together with the answers in the closed question.

The questionnaire was administered by one of the researchers who is a speech and language pathologist with expertise in dementia and a Brazilian-Portuguese native speaker. This examiner was not involved in any part of the diagnostic process of our participants, since them were already diagnosed when they were selected to participate of this study. The administration time needed for the questionnaire was about 20 min.

### Data Analysis

Pearson's Chi-Squared Test and Fisher's exact test were used to investigate an association between the first symptoms and the participants' diagnosis. Sociodemographic features of the respondents (caregivers of individuals with PPA and AD) were compared using Pearson's Chi-Squared Test and *t*-student test. A significance level of 5% was adopted.

## Results

Overall, 20 individuals with PPA and 16 with AD were included in the study. In relation to sex distribution, 50% of the patients who had PPA and 62.5% of the patients who had AD were female. In turn, the mean age of the groups was 68.1 (±7.7) years for patients with PPA and 79.9 (±9.0) for patients with AD. As for the educational level, subjects in the PPA group had an average of 13.5 (±4.3) years of study, while subjects in the group with AD had an average of 5.2 (±4.0) years of study. All participants with PPA and AD were right-handed. Descriptive data for all participants are shown in Table 1.

**Table 1:** Sociodemographic characteristics of the participants.

	**PPA (total) (*N* = 20)**	**Semantic PPA (*N* = 8)**	**Logopenic PPA (*N* = 3)**	**Non-fluent PPA (*N* = 7)**	**Non-classifiable PPA (*N* = 2)**	**AD (*N* = 16)**
Sex (F)—*N*(%)	10 (50.0)	2 (25.0)	2 (66.7)	4 (57.1)	2 (100)	10 (62.5)
Age—mean (SD±)	68.1 (7.7)	65.0 (8.5)	67.0 (9.6)	72.4 (5.9)	67.0 (2.8)	79.9 (9.0)
Age of first symptoms—mean (SD±)	63.0 (8.6)	59.7 (3.1)	64.0 (6.2)	66.4 (3.3)	63.5 (6.2)	68.8 (8.4)
Educational level—mean (SD±)	13.5 (4.3)	13.9 (3.6)	13.3 (4.6)	13.3 (5.0)	13.0 (8.5)	5.2 (4.0)
Hand dominance (right-handed)—*N* (%)	20 (100)	8 (100)	3 (100)	7 (100)	2 (100)	16 (100)
**Race—*****N*** **(%)**						
White	18 (90.0)	7 (87.5)	3 (100)	6 (85.7)	2 (100)	10 (62.5)
Mixed	2 (10.0)	1 (12.5)	0 (0)	1 (14.3)	0 (0)	3 (18.8)
Black	0 (0)	0 (0)	0 (0)	0 (0)	0 (0)	2 (12.5)
Indigenous	0 (0)	0 (0)	0 (0)	0 (0)	0 (0)	1 (6.3)

*PPA, primary progressive aphasia; AD, Alzheimer's disease; F, female; SD, standard deviation*.

The respondents of our questionnaire were the caregivers of participants with PPA and AD. Among the caregivers with PPA, 70% were female and had an average age of 52.4 (± 15) and an average education of 15.6 (± 1.5). 40% of the caregivers of patients with PPA was composed by spouses, 30% children, 5% brothers and 5% nephews. The caregivers of subjects with AD were mostly women (81.3%), with an average age of 54.1 (± 9.8) and average education of 13.4 (± 2.5). The relationship type of caregivers of people with AD were 75% children, 6.3% spouses and 12.5% son-in-law or daughter-in-law. The characteristics of these respondents were compared and there was no statistically significant difference between age (*p* = 0.70) and sex (*p* = 0.94). Education was significantly higher among caregivers of people with PPA (*p* = 0.007). The type of relationship between the caregiver and the patient were significantly different between the groups (*p* = 0.02), indicating that caregivers of people with PPA were mostly spouses and caregivers of people with AD were children.

At first, the early symptoms reported by the family members/caregivers of the patients were compared between the PPA and AD groups. A statistically significant difference was found for the symptoms of anomia (*p* = 0.00), memory difficulty (*p* = 0.00), speech motor difficulty (*p* = 0.00), and paraphasias (*p* = 0.01). Anomia, speech motor difficulty and paraphasias were predominant in the group with PPA. On the other hand, memory difficulties have been reported only in people with AD. There was no significant difference for behavioral symptoms (*p* = 0.08), agrammatism (*p* = 0.69), temporal/spatial disorientation (*p* = 0.19), executive functioning difficulties (*p* = 0.10), difficulty reading and writing (*p* = 0.69), difficulty repeating (*p* = 0.11), comprehension difficulties (*p* = 0.36), and echolalia (*p* = 0.36).

**Figure 1:**
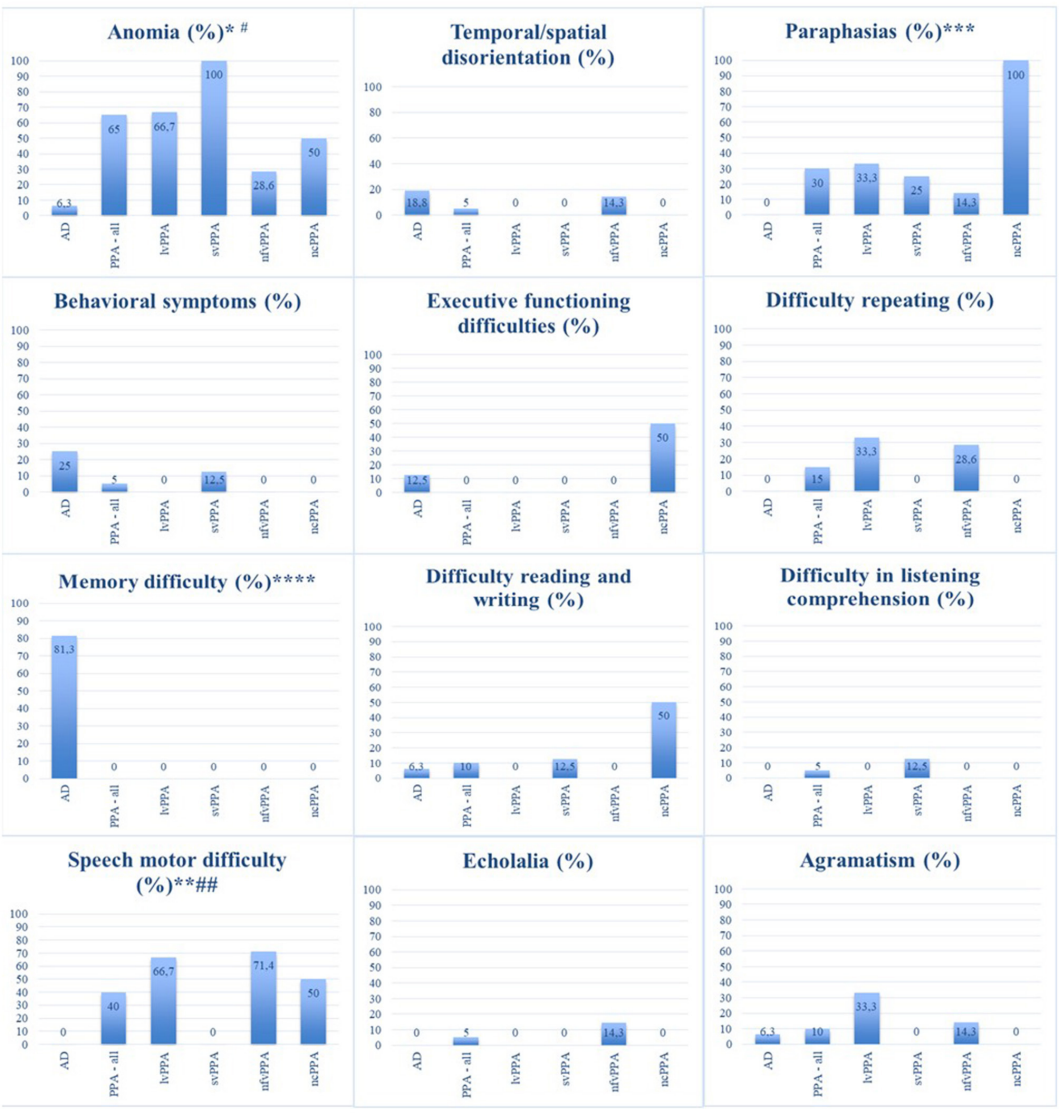
Percentages of occurrence of the first symptoms in each group of participants. AD, Alzheimer's disease; PPA, primary progressive aphasia; lvPPA, logopenic variant primary progressive aphasia; svPPA, semantic variant primary progressive aphasia; nfvPPA, non-fluent/agrammatic variant primary progressive aphasia; ncPPA, non-classifiable primary progressive aphasia. ^*^Statistically significant difference for the symptom of “anomia” (*p* = 0.00), when comparing PPA and AD groups. ^**^Statistically significant difference for the symptom of “speech motor difficulty” (*p* = 0.00), when comparing PPA and AD groups. ^***^Statistically significant difference for the symptom of “paraphasias” (*p* = 0.01), when comparing PPA and AD groups. ^****^Statistically significant difference for the symptom of “memory difficulty” (*p* = 0.00) reported only in the AD group, when comparing PPA and AD groups. ^#^Statistically significant difference for the symptom of “anomia” (*p* = 0.04) associated with a higher frequency in svPPA, when comparing PPA groups. ^##^Statistically significant difference for the symptom of “speech motor difficulty” (*p* = 0.03) associated with a higher frequency in nfvPPA, when comparing PPA groups.

When the early symptoms of the PPA variants were compared, a statistically significant difference was found for anomia (*p* = 0.04) and speech motor difficulty (*p* = 0.03), which were the most frequent in the svPPA and nfvPPA variants, respectively. There was no significant difference for behavioral symptoms (*p* = 0.66), agrammatism (*p* = 0.38), temporal/spatial disorientation (*p* = 0.58), difficulty reading and writing (*p* = 0.19), difficulty repeating (*p* = 0.31), comprehension difficulties (*p* = 0.66), and echolalia (*p* = 0.58). Symptoms related to deficits in memory and executive function were not compared, as they were reported only in the AD group. Figure 1 shows the percentage of occurrence of each symptom in each group.

## Discussion

This study suggests a potential relationship between the early symptoms reported by family members and caregivers close to individuals with PPA and AD, and the diagnosis of these diseases. The results of this study showed that the initial language symptoms, such as anomia and speech difficulties, were significantly associated with the svPPA and nfvPPA variants, respectively; while memory-related symptoms were associated with AD.

The study investigated the early symptoms through a clinical interview with the caregivers of the participants, with no neuropsychological assessment. Nevertheless, the early symptoms that were significantly associated with the svPPA, nfvPPA and AD groups were in line with the neuropsychological descriptions found in the literature (6, 7, 11, 16, 17). A broad review (6) that described the linguistic aspects and anatomical characteristics of the three PPA variants, in addition to the behavioral variant FTD, describes in detail the clinical findings in the variants, confirming that the identification by confrontation is impaired in the svPPA, as well as the articulation and speed of speech have changes in nfvPPA.

The results of this study are in line with the findings of another study (18) that investigated the symptoms of the initial stages and also the pathological analyzes of individuals with FTD, which reported that most of the patients with svPPA reported anomia as one of the first symptoms, and that memory symptoms were rarely reported in patients with frontotemporal changes, such as PPA and behavioral variant FTD. That study (18) also reported that, even in the early stages and with few manifestations, patients with FTD already had changes in the pathological analyzes, and also already reported typical symptoms of the diagnostic criteria of their variants. The authors stressed the importance of evaluating the first clinical symptoms in order to contribute to early diagnosis and favor the prognosis of these individuals.

The results of the present study also indicate that the early symptoms related to memory were significantly associated with patients with AD, while they were not reported in patients with PPA. This finding is in line with the literature, which reports that, initially, the diagnosis of AD requires that at least two cognitive domains—such as memory, learning, reasoning, behavior—must be impaired and must cause significant impairment to the individual's functionality (8, 9).

In this study, the lvPPA was not found to be associated with any initial symptoms described by the sample of participants. This may be explained by three possible reasons. First, it would be due to the fact that the difficulty repeating (1), which is one of the main characteristics of lvPPA, is difficult to perceive and observe by patients and their caregivers, since repetition is not used in common way, and is usually more detectable at a neuropsychological or speech/language examination. Second, the lvPPA is the most recently described variant and there are reports in the literature of the poor reliability of identifying its clinical characteristics (19, 20). The core clinical characteristics of the diagnostic criteria for this variant include symptoms that are not unique to it, such as anomia (which is an important symptom of svPPA) and the difficulty in repeating sentences, which is a symptom that can occur in this variant as well, despite not being one of the diagnostic criteria of nfvPPA (2). In this sense, it was possible to notice that the percentage of occurrence of repetition difficulties in our sample was similar between the lvPPA and nfvPPA groups. Thus, and as already discussed before (11), there may be questions about the diagnostic criteria used in PPAs, and reviewing these criteria may be important for more accurate diagnoses. Butts et al. (11) reported that 31% of a sample of subjects with PPA was not classifiable by the quantitative application of the current diagnostic consensus criteria (2011) (1), which is in line with Senaha and colleagues (2013) (2), who also reported such difficulties. A recent study (18) also raised the question that, although there are initial symptoms that are referred by several patients, they are not considered diagnostic criteria by the current consensus. This discussion supports the importance of studies in speakers of other languages, in order to investigate the profile and the occurrence of linguistic manifestations specific to the pattern of each language. Furthermore, the limited sample size of the present study would be the third reason to explain why an association of early symptoms with lvPPA may not have been found.

The first symptoms that were unique in certain groups of this study can be seen as red flags specifically for directing the clinical interview with the patient and caregiver, allowing for a better direction of the investigation. On the other hand, our study did not show a significantly higher occurrence of several other symptoms in any of the groups analyzed. Although the literature indicates the occurrence of these symptoms in the studied disorders, such as the occurrence of behavioral symptoms as inaugural symptoms in PPA (21), this characteristic might not be a red flag of the interview with the patient and the caregiver, despite being extremely relevant for the clinical management.

The analysis and investigation of the first symptoms presented by individuals with PPA is essential for the differential diagnosis of the disorder and for the classification of its variants. Studies that described the concept and diagnostic criteria of PPA (1, 5) emphasize that language deficits must be the main aspects with changes in order to define it as a case of progressive aphasia, while other cognitive domains, such as memory, visuospatial skills, and behavior should remain without significant changes, at least in the first two years of the disease (5). Thus, the identification of the early symptoms that are prevalent in the language domain is essential for a more accurate diagnosis of PPA.

When analyzing the Brazilian context in which this study was carried out, it is known that the diagnosis of patients with PPA is not always carried out accurately, and mistakes and diagnostic errors may occur due to the delay for the patient to reach the reference services (10, 12). Therefore, it is clear that a retrospective investigation of the early symptoms has a more relevant role in this scenario, stressing that a thorough clinical interview should be a priority in monitoring the suspected PPA in this population, contributing to the accuracy of the diagnosis.

All participants in this study were Brazilian Portuguese speakers. However, most scientific knowledge related to PPA is obtained from studies with English speakers. As the PPA is a syndrome centered on language impairment, it is important to emphasize studies that aim to characterize the profile and linguistic manifestations of speakers of other languages, especially in Latin languages, such as Portuguese (10). The unique characteristics of different languages have different perspectives on the development, plasticity and cognitive reserve of specific linguistic networks and, thus, could have different diagnostic criteria, which would apply for each language specifically (16).

It is important to note that the interpretation of our results have to be made considering three important limitations: (I) The sample size of our study, especially the small number of participants with lvPPA. We believe that the results of the lvPPA group are not conclusive due to the sample size. However, we believe it was important not to exclude them from the study in order to call attention to one of the great difficulties to conduct research with this profile of participants in Brazil, which is collecting significant samples from patients with PPA due to the inaccurate and late detection and diagnosis (12). (II) The heterogeneity of sociodemographic characteristics between groups. Participants with PPA and also their caregivers had higher educational level than their peers of the group with AD. Individuals with higher education and better socioeconomic status may tend to seek medical attention sooner in the face of milder and lesser known symptoms, such as the language symptoms of PPA. The caregivers may also notice and report symptoms more accurately. For reasons of study feasibility, most patients with PPA included in this study were from private health services, while those with AD were from public health institutions. As the diagnosis of AD is better known and more easily performed, this type of dementia has a higher frequency of detection in the Brazilian public health system to the detriment of cases of PPA. In turn, although the sample of this study cannot be considered large or expressive, given the reality of Brazilian scientific production on this topic, which is scarce and basically reduced to case studies (10), it can be considered a reasonable sample. (III) Not all participants could perform functional neuroimaging or cerebrospinal fluid examination with dosage of AD biomarkers, due to constraints and because such tests are not covered by the Brazilian public health system.

Finally, the investigation and knowledge of the first symptoms presented by patients with PPA has great potential to assist in the differential diagnosis of the disease variants. This study showed that the symptoms of “anomia” and “speech motor difficulties” are the most frequently reported in svPPA and nfvPPA, respectively; while the symptoms associated with memory are more often related to AD. Further research with larger and even more representative samples may contribute to the description of the profile and clinical symptoms presented by Brazilian Portuguese-speaking patients with PPA.

## Data Availability Statement

The raw data supporting the conclusions of this article will be made available by the authors, without undue reservation.

## Ethics Statement

The studies involving human participants were reviewed and approved by the committee of the Irmandade Santa Casa de Misericórdia de Porto Alegre (ISCMPA) and Universidade Federal de Minas Gerais (HC-UFMG). The patients/participants provided their written informed consent to participate in this study.

## Author Contributions

TR: conceived the idea, designed and carried out the study, took lead in writing the manuscript. TM, PC, FS, and LF: contributed to the recruitment of participants and the final manuscript. BB: conceived the idea, designed the study, supervised the study, and reviewed the final manuscript. All authors contributed to the article and approved the submitted version.

## Conflict of Interest

The authors declare that the research was conducted in the absence of any commercial or financial relationships that could be construed as a potential conflict of interest.
