# Supplementary material for: First Symptoms of Primary Progressive Aphasia and Alzheimer's Disease in Brazilian Individuals
Source: Front Neurol. 2021 Jun 11;12:628406. doi: 10.3389/fneur.2021.628406 (PMC8226014; doi:10.3389/fneur.2021.628406)
Supplement: Supplementary file 1 [file Table_1.pdf]

Questionnaire about first symptoms of people with primary progressive aphasia and Alzheimer's disease – English Version

**1. Personal and sociodemographic data**<br>*(These data can be obtained from the medical records and then confirmed by the caregiver)*
1.1 Patient
Name:
Sex: ( ) Female ( ) Male
Age:
Years of Education:
Race: ( ) White ( ) Mixed ( )Black ( ) Indigenous ( ) Yellow
Hand-Dominance: **( )** Right-handed ( ) Left-handed
1.2 Caregiver
Name:
Sex: ( ) Female ( ) Male
Age:
Years of Education:
Race: ( ) White ( ) Mixed ( )Black ( ) Indigenous ( ) Yellow
Relationship with the patient:
**2. Clinical data of patient**<br>*(These data can be obtained from the medical records and then confirmed by the caregiver)*
Neurological Diagnosis:<br>( ) Non-fluent PPA ( ) Semantic PPA ( ) Logopenic PPA<br>( ) Non-classifiable PPA ( ) Alzheimer's disease
Age of first symptoms:
**3. Please, describe what were the first symptoms or difficulties that your family member (or person that you care) had at the very beginning of this disease?**<br>*(This question should be asked directly to the patient's caregiver and allow sufficient time for the respondent to describe the symptoms with his or her words)*<br>____<br>____<br>____<br>____<br>____
**4. I will give you some symptoms, and you can answer affirmatively to those that your relative (or person that you care) presented at the very beginning of this disease:**<br>*(You might rephrase the sentences and/or give examples if you think the respondent did not understand the symptom)*<br>( ) Difficulty to remember the name of things or people, or to recognize them<br>( ) Exchange of a word for another<br>( ) Repetition of the same stories or questions<br>( ) Difficulty to understand what other people say<br>( ) Difficulty in articulating words (slurred speech, effort, stuttering, distortions or substitutions of sounds in the words, or slow speech)<br>( ) Difficulty in temporal (knowing the time or day) and/or spatial orientation (knowing where the person are)<br>( ) Changes in behavior, such as isolation, irritability, impulsivity, inappropriate sexual behaviors, changes in food preferences or behaviors, sadness, apathy

(   ) Difficulty in repeating words or sentences heard<br>(   ) Difficulty in organizing the words in the sentence, or in using tense verbs, prepositions and articles.<br>(   ) Difficulty in reading and writing.<br>(   ) Difficulty in planning and organizing routine and activities as well as in reasoning.<br>( ) Did you remember some other difficulty that were not mentioned here? If yes, what was the difficulty?

Questionário sobre os primeiros sintomas de pessoas com afasia progressiva primária e doença de Alzheimer – Versão em Português

**1. Dados pessoais e sociodemográgicos**<br>*(Estes dados podem ser obtidos dos prontuários médicos e depois confirmados com o cuidador)*
1.1 Paciente
Nome:
Sexo: (   ) Feminino (   ) Masculino
Idade:
Anos de escolaridade:
Raça: (   ) Branco   (   ) Pardo   (   )Preto   (   ) Indígena   (   ) Amarelo
Dominância Manual: (   ) Destro   (   ) Canhoto
1.2 Cuidador
Nome:
Sexo: (   ) Feminino (   ) Masculino
Idade:
Anos de escolaridade:
Raça: (   ) Branco   (   ) Pardo   (   )Preto   (   ) Indígena   (   ) Amarelo
Relação com o paciente:
**2. Dados clínicos do paciente**<br>*(Estes dados podem ser obtidos dos prontuários médicos e depois confirmados com o cuidador)*
Diagnóstico neurológico:<br>(   ) APP não-fluente   (   ) APP semântica   (   ) APP logopênica<br>(   ) APP não-classificável   (   ) Doença de Alzheimer
Idade dos primeiros sintomas
**3. Por favor, descreva com as suas palavras quais foram os primeiros sintomas ou dificuldades que seu familiar (ou pessoa que você cuida) teve bem no início desta doença.** *(Esta pergunta deve ser feita diretamente ao cuidador do paciente e permitir tempo suficiente para que o respondente descreva os sintomas com as suas palavras)*<br>____<br>____<br>____<br>____<br>____
**4. Irei te dizer alguns sintomas e você deve responder afirmativamente para aqueles que seu familiar (ou pessoa que você cuida) apresentou bem no início da doença:**<br>*(Você pode parafrasear a frase e/ou oferecer exemplos se você achar que o respondente não entendeu algum dos sintomas)*<br>(   ) Dificuldade para lembrar o nome das coisas ou pessoas, ou para reconhece-los.<br>(   ) Trocas de uma palavra por outra

( ) Repetição das mesmas histórias ou perguntas
( ) Dificuldade para entender o que as outras pessoas falam.
( ) Dificuldade para articular as palavras (como fala arrastada, com esforço, gagueiras, distorções ou substituições de sons nas palavras, ou fala lentificada)
( ) Dificuldade para se orientar no tempo (saber a hora ou dia) e/ou no espaço (saber em que lugar está)
( ) Mudanças de comportamento, como isolamento, irritabilidade, impulsividade, comportamentos sexuais inapropriados, mudanças nas preferências ou comportamento alimentares, tristeza, apatia
( ) Dificuldade para repetir frases ou palavras ouvidas
( ) Dificuldade para organizar as palavras dentro da frase, conjugar verbos, usar preposições e artigos
( ) Dificuldade para ler e escrever
( ) Dificuldade para planejar e organizar sua rotina e atividades, assim como para raciocinar
( ) Você lembrou se mais alguma outra dificuldade que eu não mencionei aqui? Se sim, qual foi a dificuldade?
